# *Origanum majorana* L*.* protects against neuroinflammation-mediated cognitive impairment: a phyto-pharmacological study

**DOI:** 10.1186/s12906-023-03994-x

**Published:** 2023-05-20

**Authors:** Reham Wagdy, Reham M. Abdel-Kader, Ahmed H. El-Khatib, Michael W. Linscheid, Heba Handoussa, Nabila Hamdi

**Affiliations:** 1 Department of Pharmaceutical Biology, German University in Cairo, Cairo, Egypt; 2 Department of Pharmacology and Toxicology, Faculty of Pharmacy and Biotechnology, German University in Cairo, New Cairo City, 11835 Egypt; 3 Department of Pharmaceutical Analytical Chemistry, Faculty of Pharmacy, Ain Shams University, Cairo, Egypt; 4 Department of Chemistry, Humboldt-Universität Zu Berlin, Berlin, Germany

**Keywords:** *Origanum majorana*, Cognition, Neuroinflammation, Phenolics, LPS, Neurodegeneration

## Abstract

**Background:**

Neuroinflammation and oxidative stress are critical players in the pathogenesis of numerous neurodegenerative diseases, such as Alzheimer’s disease (AD) which is responsible for most cases of dementia in the elderly. With the lack of curative treatments, natural phenolics are potential candidates to delay the onset and progression of such age-related disorders due to their potent antioxidant and anti-inflammatory effects. This study aims at assessing the phytochemical characteristics of *Origanum majorana* L. (OM) hydroalcohol extract and its neuroprotective activities in a murine neuroinflammatory model.

**Methods:**

OM phytochemical analysis was done by HPLC/PDA/ESI-MS^n^. Oxidative stress was induced in vitro by hydrogen peroxide and cell viability was measured using WST-1 assay. Swiss albino mice were injected intraperitoneally with OM extract at a dose of 100 mg/kg for 12 days and with 250 μg/kg LPS daily starting from day 6 to induce neuroinflammation. Cognitive functions were assessed by novel object recognition and Y-maze behavioral tests. Hematoxylin and eosin staining was used to assess the degree of neurodegeneration in the brain. Reactive astrogliosis and inflammation were assessed by immunohistochemistry using GFAP and COX-2 antibodies, respectively.

**Results:**

OM is rich in phenolics, with rosmarinic acid and its derivatives being major constituents. OM extract and rosmarinic acid significantly protected microglial cells against oxidative stress-induced cell death (*p* < 0.001). OM protected against the LPS-induced alteration of recognition and spatial memory in mice (*p* < 0.001) and (*p* < 0.05), respectively. Mice that received OM extract prior to the induction of neuroinflammation showed comparable histology to control brains, with no overt neurodegeneration. Furthermore, OM pre-treatment decreased the immunohistochemistry profiler score of GFAP from positive to low positive and COX-2 from low positive to negative in the brain tissue, compared to the LPS group.

**Conclusion:**

These findings highlight the potential preventive effects of OM phenolics against neuroinflammation and pave the way toward drug discovery and development for neurodegenerative disorders.

## Background

Alzheimer’s disease (AD) is a complex neurodegenerative disease, with neuroinflammation being a significant contributor [1]. Activated microglia and astrocytes overexpressing pro-inflammatory cytokines were described a long time ago surrounding neurofibrillary tangles early during their formation [2, 3]. Pro-inflammatory cytokines, such as tumor necrosis factor-alpha (TNF-α) and interleukin-6 (IL-6), were elevated in both serum and brain tissue of AD patients and their levels correlated with disease progression [4–6]. The sustained activation of microglia results in the release of potentially neurotoxic pro-inflammatory cytokines and free radicals [7, 8]. Moreover, the abnormal activation and proliferation of astrocytes results in astrogliosis, another pathological hallmark of AD. In fact, astrogliosis was observed in the very early stages of AD around amyloid beta (Aβ) plaques and correlated with their density [9, 10].

Toll-like receptors (TLRs) are essential elements of innate immunity that sense pathogen- and danger-associated molecular patterns (DAMPs) involving Aβ. TLR4 is highly expressed on microglia, and its contribution to AD was demonstrated in several studies. An increased TLR4 expression and its downstream pro-inflammatory cytokines were found in the frontal cortex of AD patients [11]. Intracerebroventricular injection of Aβ in wild-type but not in TLR4 knockout mice caused an inflammatory response resulting in synaptic damage, neuronal loss, and decline in cognitive function [12]. DAMPs produced from necrotic or hyper excitatory neurons induce neurite degeneration via TLR4 by activating MAP kinases [13]. Interestingly, Zhao and colleagues reported for the first time the presence of bacterial lipopolysaccharide (LPS) in the lysates obtained from several brain regions (hippocampus and neocortex) of AD patients, highlighting the implication of the gastrointestinal tract microbiome-derived LPS as a significant internal contributor to neuroinflammation [14]. LPS from Gram-negative bacteria has an immunostimulatory effect by activating TLR4 pathway with the expression of many inflammatory mediators [15]. Various LPS models have been used as neuroinflammatory experimental models [16] among them the daily intraperitoneal injection (i.p.) of LPS that we and others demonstrated to induce neurodegeneration, cognitive impairment, and Aβ1-42 accumulation [17–20]. These LPS models are helpful to assess strategies that aim to modulate neuroinflammation to delay or halt the progression of neuroinflammtion-related neurodegeneration. Dietary phenolics are potential candidates for such strategies based on their antioxidant and anti-inflammatory properties [21].

*Origanum majorana* L. (OM), commonly known as sweet marjoram, is a perennial evergreen subshrub commonly found in the Mediterranean region and used extensively in folk medicine for asthma and headache. OM is rich in phenolics and flavonoids [22] with promising pharmacological properties such as antioxidant and acetylcholinesterase inhibitory activities [23, 24]. In vitro*, Origanum* inhibited pro-inflammatory cytokines such as IL-6, NO and TNF in LPS-stimulated macrophages [25], provided anti-inflammatory activity and facilitated wound healing in human keratinocytes [26]. Furthermore, several in vivo studies support the promising protective effects of *Origanum* in various models of inflammation and oxidative stress, including gastritis, cardiotoxicity, and LPS-induced endotoxemia models [27–29]. Focusing on neuroinflammation and neurodegeneration, a recent study showed strong antioxidant properties of *Origanum majorana* that protected neurons from haloperidol-induced cognitive and motor deficits by decreasing oxidative stress [30]. Moreover, rosmarinic acid, a major phytochemical in OM extract, could mitigate LPS-induced cognitive deficits, neurodegeneration, and the overproduction of proinflammatory cytokines in the hippocampus and cortex and reverse the oxidant-antioxidant balance in a rat model of LPS-induced neuroinflammation [31]. Based on this solid evidence of antioxidant and anti-inflammatory activities in relevant models, OM was chosen in this study to analyze its phytochemical composition and to assess its possible neuroprotective role in an LPS-induced neuroinflammatory mouse model concerning neurodegeneration, cognitive impairment, astrogliosis and inflammatory mediators.

## Methods

### Plant material

OM fresh leaves were collected from El-Orman Botanical Garden, Giza, Egypt. Engineer Therese Labib, the consultant at El-Orman Botanical Garden and the National Gene Bank at the Ministry of Agriculture, Egypt, kindly authenticated the plant. A voucher specimen of OM numbered (PHBL-00324) was deposited at the Pharmaceutical Biology Department at the German University in Cairo (GUC). No permission for plant collection was required, as OM is not an endangered species or at risk of extinction. Air-dried powdered OM leaves (2 kg) were extracted for 2 h using 5 L of distilled water at 60 to 70 °C to extract all polar compounds. The obtained extract was subjected to filtration and evaporation under reduced pressure in vacuo (BUCHI, Rotavapor, R-210; Switzerland) until complete dryness. The obtained dried residue (150 g) was extracted with purified 90% ethanol (Sigma-Aldrich, Darmstadt, Germany). Afterward, the ethanolic extract was subjected to filtration and evaporation under reduced pressure in vacuo. The residue was then washed with acetone (Sigma-Aldrich, Darmstadt, Germany), and lyophilization was done to produce the crude phenolic content (28 g) [32]. Isolated and purified rosmarinic acid (RA) was obtained from another study conducted by our research group [20, 33].

### Determination of total phenolic and flavonoid content

Folin-Ciocalteu method was conducted to calculate the phenolic content of OM extract. It was measured as mg gallic acid equivalent (GAE)/mg extract [34]. Briefly, OM extract of 10 mg/ml in methanol (Sigma-Aldrich, Germany) and serial dilutions of Gallic acid standard (0 to 200) µg/ml were prepared. Afterward, 200 µl of each prepared sample was transferred to a 2 ml tube, followed by the addition of 1 ml Folin-Ciocalteu reagent and left to stand for 5 min at room temperature. Next, 800 µl sodium carbonate solution (7.5%) (El-Nasr Pharmaceuticals Co., Egypt) were added and left for 2 h at room temperature in the dark, and then the absorbance was measured at 750 nm (Perkin Elmer, Waltham, MA, USA). Preparation of the blank was done using a similar protocol. Serial dilutions of Gallic acid standard (Sigma-Aldrich, Germany) were used for the calibration curve. The flavonoid content was estimated according to the method of Schofield, Mbugua, and Pell as mg quercetin equivalent QE/mg extract [35]. OM extract of 0.4 mg/ml in methanol was prepared. In a 96-well plate, 150 µL of OM extract was added to 100 µL of 2% (w/w) AlCl_3_ (El-Nasr Pharmaceuticals Co., Egypt)**.** Same experimental steps were performed for the standard quercetin (Sigma-Aldrich, Germany) (0–80 µg/ml). All samples were then left for 30 min, including the blank, at room temperature. A microplate reader (Perkin Elmer, Waltham, MA, USA) was used to measure the absorbance at 430 nm.

### Phytochemical characterization

In order to profile the active metabolites in OM active extract, high-performance liquid chromatography HPLC/PDA/ESI-MS^n^ was applied using HPLC Agilent 1200 series instrument provided with high-performance autosampler, binary pump, and PDA G 1290 C (SL; Agilent Technologies, Waldbronn, Germany). For analytes separation, acquity BEH C18 100 mm × 2.1 mm column (particle size, 1.7 μm) was used with RP C18 100 A° guard column with dimensions (5 × 3 mm, i.d.; 5 µm). A mobile phase gradient of 2% acetic acid in purified water (A) and 90% methanol in purified water (B) was implemented. The flow rate operation of the mobile phase was adjusted at 50 µL/min. OM extract was solubilized in 5% methanol and 2% acetic acid at 1 mg/mL concentration and filtered with a 0.2 µm syringe filter. The gradient was as follows: 0 to 60 min, 5% B; 60 to 70 min, 50% B; 70 to 80 min, 90% B; and 80 to 90 min, 5% B. The volume of the injected sample was 10 µL. A Fourier transform ion cyclotron resonance mass analyzer was supported with an Electrospray ionization (ESI) system. The mass was evaluated in Fourier Transform Ion Cyclotron Resonance (FT-ICR) in the full scan and in trap in Mass/Mass (MS/MS) mode (fragmentation). X-calibur® software was used for system control and data acquisition. MS scan and detection was applied in the negative ion mode with the followings: capillary voltage of 36 V, temperature of 275 °C, API source voltage of 5 kV, desolvation temperature of 275 °C and the nitrogen was used as desolvation and cone gas at a flow rate of 15 L/min. The entire run duration was 89 min. The mass range covered was from 150 to 2,000 m/z and resolving power to 100,000 [20].

### Antioxidant effect of OM hydroalcoholic extract and rosmarinic acid

BV2 mouse microglial cell lines were cultured in 96-well plates, for 24 h, at a density of 10,000/well in Roswell Park Memorial Institute medium (RPMI; Lonza, Belgium) containing 10% fetal bovine serum (Lonza, Belgium) and 1% penicillin–streptomycin (Lonza, Belgium). The safety of OM extract and RA was tested by adding increasing concentrations of OM (25, 50, 100, 200, 400 µg/ml) or RA (5, 10, 20 µg/ml) to the cells for 72 h. WST-1 cell viability assay kit (Takara, Canada) was used to evaluate the cell viability, and absorbance was measured at 450 nm by a microplate reader (Perkin Elmer, Waltham, MA, USA). In another experiment, oxidative stress was induced by incubating BV2 cells with hydrogen peroxide (H_2_O_2_) (Sigma Aldrich) for 24 h at increasing concentrations (50, 100, 150, 200, 250, 300, 350 µM) to determine its IC_50_. The antioxidant effect on BV2 cells was assessed by pre-treating the cells with different concentrations of OM or RA for 24 h prior to the stimulation with H_2_O_2_ at IC_50_, followed by the measurement of cell viability.

### Animals

Healthy adult male Swiss albino mice aged 2 to 3 months weighing 20 to 30 g were purchased from the National Institute of Research animal facilities, Cairo, Egypt. The weight of the mice was recorded daily for an accurate measurement of the treatment dose. Mice were acclimatized for one week at the GUC animal house at room temperature under a 12-h light/12-h dark cycle, with access to food and water ad libitum. Mice were first subjected to behavioral tests; then brains were harvested for further research and analysis. In order to avoid any interference of other chemical compounds with the scientific outcomes of the study, the use of anesthetics was avoided based on their previously reported effects on memory and cognition [36]. The cervical dislocation was chosen as a physical method for rapid sacrifice [37] that was shown to be completely safe on the brain [38]. Cervical dislocation was applied by a trained operator to achieve the highest level of animal welfare. Animal care and treatments were conducted according to the National Research Council's Guide for the Care and Use of Laboratory Animals and in accordance with ARRIVE guidelines. The study was approved by the Research Ethics Committee at the German University in Cairo (project ID 2015–04-HH). This study was randomized and blinded whenever possible. Due to the color of the given plant extract, the experimenter could not be blinded to the drug allocation. Randomization was carried out using Graphpad online random number generator.

### LPS mouse model

The neuroinflammatory mouse model used in the current study was induced by daily i.p. injection of 250 µg/kg/day LPS for 7 successive days. This model was previously shown to induce oxidative stress, neuroinflammation, neurodegeneration and memory impairment [17, 20, 39]. The experimental unit used was a single animal, where mice were randomly divided into four experimental groups of eight mice each (without exclusion) [17, 20]. The total number of mice (32) in the study was decided based on previous studies using the same neuroinflammatory model [17]. LPS (Sigma Aldrich, Strain: *Escherichia coli*, 055:B5) was dissolved in saline and injected i.p at 250 µg/kg/day. OM extract was dissolved in saline and injected i.p at 100 mg/kg/day, based on previous work from our group [20, 40]. Mice were treated for 12 consecutive days: the control group received saline only, the LPS group received LPS starting day 6, the LPS + OM group received OM extract starting day one and LPS starting day 6, and the OM group received OM extract for 12 consecutive days [41]. Any two subsequent injections on the same day were separated by 2 h.

### Behavioral tests

In this study, memory and cognitive functions were considered the primary outcomes and were assessed on day 12. All mice were subjected to both Y-maze and novel object recognition tests, and blinding was applied during testing and analysis of the results. The testing followed a randomized order of the tested mice.

#### Novel object recognition

This test was carried out as previously described [42]. The test apparatus is an empty wooden box attached to an elevated camera to afford a full image of the arena. Transportation of the mice to the experimental room was performed 2 days before the test day for acclimatization (60 min). Mice were permitted to habituate for 10 min in the empty arena a day prior to the test day. In the training phase, each mouse was given 10 min to explore the box with two identical objects, either cubes or pyramids. After 2 h, one of the familiar objects was switched with a novel object in the testing phase and mice were left to freely explore both objects for 5 min. The videos were recorded and analyzed to calculate the discrimination ratio as the time spent by the mouse exploring the novel object divided by the exploration time of both objects. Mice with intact memory are expected to explore the novel object longer than the familiar one. The pyramids and the cubes were randomly used as ‘the novel objects’ to avoid bias.

#### Y-maze spontaneous alternation test

To evaluate the spatial working memory, Y-maze test was conducted as described previously [43]. The test apparatus has 3 arms that are equally distanced, extending from a central platform at 120°. Transportation of the mice to the experimental room was performed 2 days before the test day for acclimatization (60 min). During the test, each mouse was positioned in the center of the Y-maze to explore it for 8 min. The sequence of arm entries was manually recorded, the entry of three consecutive arms represented one alternation, and alternations were only considered when the mouse entered three different arms consecutively without repetitions. Mice with intact memory tend to explore the three arms equally with no repetitions. The spontaneous alternation percentage was calculated using this formula: [number of actual alternations/total number of entries-2] × 100. A higher spontaneous alternation percentage indicates a better memory.

### Histopathology

Brains were fixed and processed using 10% formalin within 24 h. Subsequently, fixed brains were washed by water and were dehydrated using serial dilutions of alcohol. Paraffin-embedded samples were sectioned at 4–5 µm thickness using sledge microtome. Tissues were later deparaffinized to be stained with hematoxylin and eosin (H&E) (VMR, Darmstadt, Germany).

### Immunohistochemistry

Brain sections of 5 µm thickness were moved onto poly-lysine-coated slides and were deparaffinized using xylene. Next, they were boiled in 10 mM citrate buffer (pH 6.0) and cooled to room temperature. The endogenous peroxidase was blocked by hydrogen peroxide for 10 to 15 min. The sections were incubated with primary antibodies against cyclooxygenase 2 (COX-2; 1:50) (Invitrogen, Karlsruhe, Germany) or glial fibrillary acidic protein (GFAP; 1:50) (Invitrogen, Karlsruhe, Germany) for 60 min; then rinsed with phosphate-buffered saline (Lonza, Belgium) four times. The sections were treated with biotinylated secondary antibodies for 10 min followed by the addition of streptavidin peroxidase (Carl Roth, Karlsruhe, Germany) for another 10 min, and then visualized with peroxidase-compatible chromogen (3, 3'-diaminobenzidine) DAB mixture (Sigma Aldrich, Munich, Germany).

### Statistical analysis

Statistical analysis was conducted using the one-way analysis of variance (ANOVA) followed by Tukey's multiple range tests and expressed as mean ± SEM (standard error of mean). GraphPad Prism (version 7.00) was used for analysis. A probability of *P* value < 0.05 was considered statistically significant (***P < 0.001, **P < 0.01, *P < 0.05). The immunohistochemistry (IHC) results were analyzed using the open-source plugin IHC profiler for fully automated digital image processing [44]. After proper separation of the DAB color spectra by the spectral deconvolution method, pixels were counted, and their percentage contribution was calculated according to pixel intensity. A semi-quantitative IHC profiler score (high positive, positive, low positive or negative) was then automatically generated. To calculate the exact number of “IHC optical density score” (from 1 to 4), the following algebraic formula was used based on IHC profiler percentage contribution (Seyed Jafari & Hunger, 2017). IHC optical density score = [(Percentage contribution of high positive × 4) + (Percentage contribution of positive × 3) + (Percentage contribution of low positive × 2) + (Percentage contribution of negative × 1)] ÷ 100.

## Results

### Total phenolic and flavonoid content

The total phenolic and flavonoid content of OM hydroalcoholic extract was 264.02 µg of GAE/mg extract and 4.37 µg of QE/mg, respectively.

### Phytochemical analysis

**Fig. 1:**
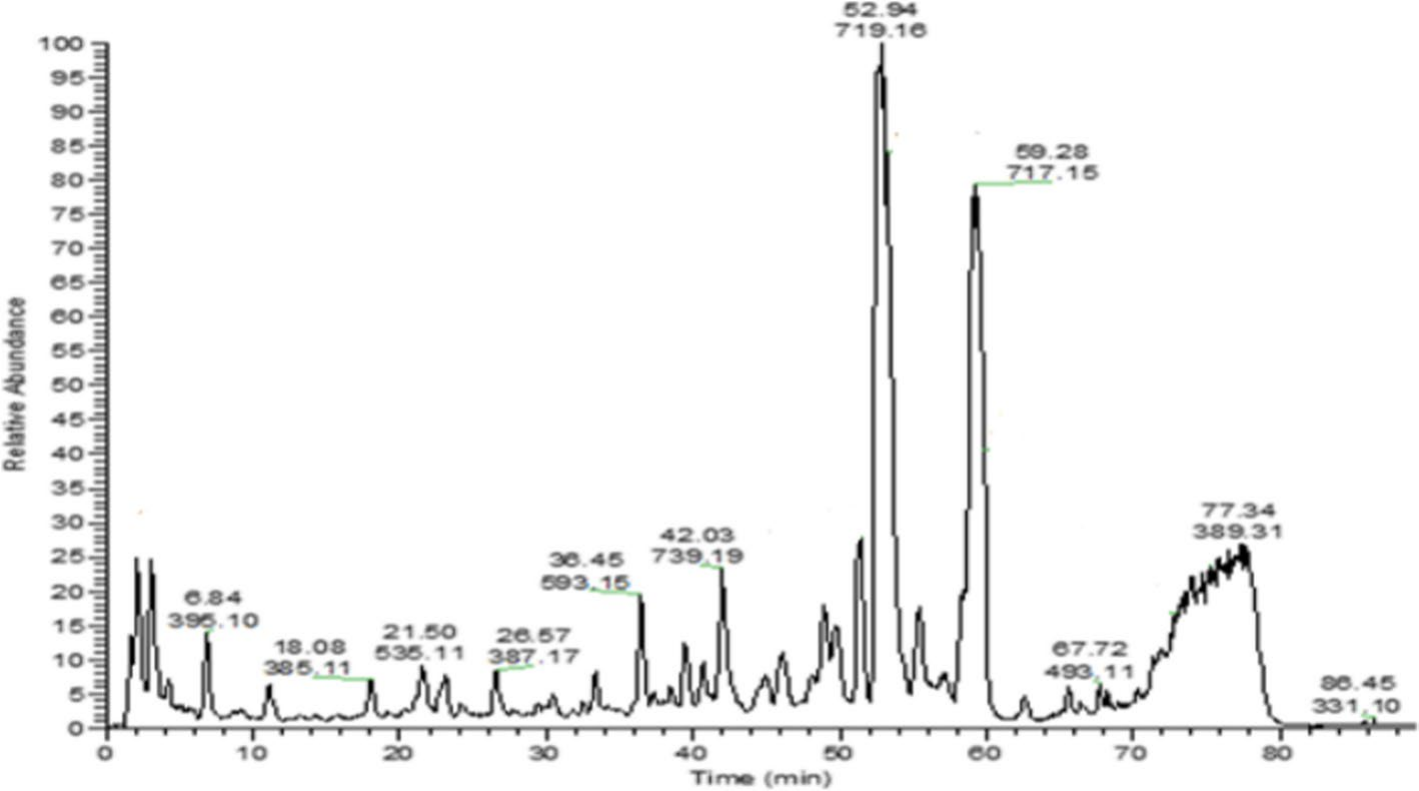
HPLC/PDA/ESI/MS^n^ of metabolites detected in hydroalcoholic extract of leaves of *Origanum majorana* L. (negative mode)

The structure of each compound was identified depending on its retention time in reverse phase, UV–Vis spectrum, and the obtained MS spectra by applying various fragmentation energies. ESI–MS analysis was conducted using the negative ion mode for better detection of phenolic molecules (Fig. 1). Table 1 summarizes the compounds detected in OM extract in increasing order of their retention time, and their identification was confirmed by using previous data published in the literature (Table 1). The profiling showed a vast array of phenolics in the extract. OM11 was identified as rosmarinic acid and its derivative (OM10) as sagerinic acid, the major compound with a peak area of 7.2% of the total chromatogram peaks’ area. RA hexoside (OM8), RA dihexoside (OM9), caffeoyl RA (OM13), and salvianolic acids (OM12, OM15 and OM16) were also found with OM15 (salvianolic acid B) representing the second-greatest peak area (6.8%). Phenolic lignin (OM6), phenolic acid (OM4), and some flavonoids (OM1 and OM7) were also identified.

**Table 1:** Peaks assignment using HPLC/PDA/ESI/MS^n^ of metabolites detected in hydroalcoholic extract of *OM* L. (negative mode)

Peak # (Compound)	Identified Compounds	Retention time (min)	UV–Vis (λmax)	[M-H]^−^(*m/z*)	Fragment ions (m/z)	Peak area %	Reference
OM1	Trihydroxyflavanone-*O*-deoxyhexosyl-*O*-hexoside	3.07	260, 330	579.15	271, 269, 313, 417	2.6	[45]
OM2	1-Hexanol-pentosylhexoside	6.84	278	395.10	350, 274.8, 230, 202/313	1.8	[46]
OM3	Salvianolic acid C derivative	11.01	320	715.17	491, 311, 179	1.2	[47]
OM4	Dihydroxyphenyllactoyl tartaric acid	17.21	326	329.12	293	1.56	[48]
OM5	Dicaffeoyl hexose	21.50	280	535.11	359,179	1.48	Tentative
OM6	Medioresinol	26.57	325	387.17	207,179	1.92	[49]
OM7	Quercetin-*O*-rhamnosyl-hexoside ( Rutin)	33.37	350, 254	609.15	1219,480,479,463, 301,179	1.52	[50]
OM8	Rosmarinic acid hexoside	45.74	320	521.13	359, 197, 179, 162	2.02	[47]
OM9	Rosmarinic acid dihexoside	48.93	320	683.16	521, 341, 179	2.3	[51]
OM10	Sagerinic acid	52.94	282, 326 (sh)	719.16	539, 521, 359, 197,179	7.2	[47]
OM11	Rosmarinic acid	53.77	280, 325 (sh)	359.08	197, 179, 161	3.82	[47]
OM12	Lithospermic acid A isomer	55.37	278, 324 (sh)	537.10	493, 359, 313, 295,179	1.72	[47]
OM13	Caffeoyl rosmarinic acid	57.12	282, 326 (sh)	538.11	359, 179	1.42	Tentative
OM14	Rosmarinic acid derivative	58.53	280, 327 (sh)	445.08	359,179	2.7	Tentative
OM15	Salvianolic acid B isomer (lithospermic acid B)	59.28	286, 324 (sh)	717.15	537, 519, 493, 359, 339, 321, 295, 197, 179	6.8	[47]
OM16	Salvianolic acid A	67.72	276, 338 (sh)	493.11	359, 313, 295, 269, 179	0.78	[47]
OM17	Myricetin methyl ether	86.45	254, 362	331.10	315, 300, 179, 151	0.84	[52]

### In vitro antioxidant activity of OM extract and RA

**Fig. 2:**
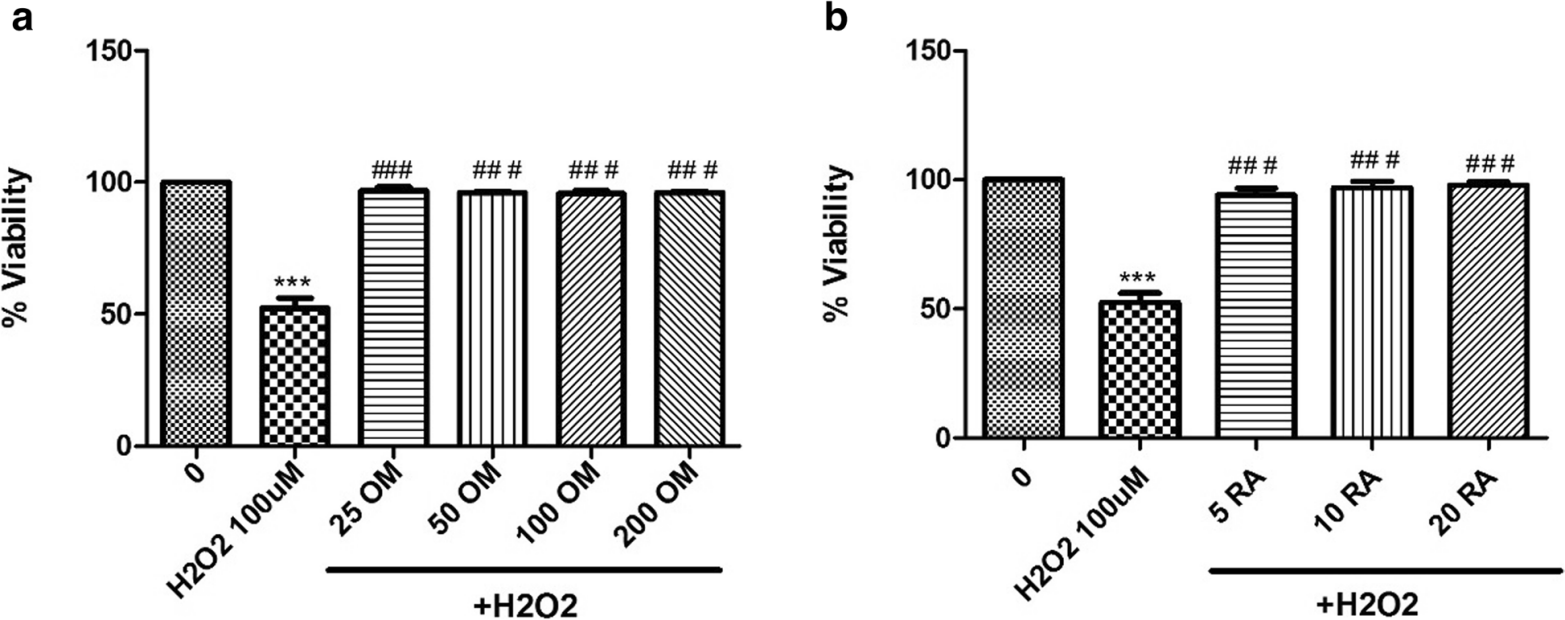
Antioxidant effect of OM extract and RA. BV2 cells were pre-treated with OM extract (**a**) or RA (**b**) using several safe concentrations for one day prior to H_2_O_2_ (IC_50_) stimulation at 100 µM. OM extract and RA protected BV2 cells from H_2_O_2_-induced cell death. Data are represented as mean ± SEM (*n* = 3). Significance from control is represented as: *** at *p* < 0.001. Significance from 100 µM H_2_O_2_ is represented as: ### at *p* < 0.001

BV2 cells pre-treated with safe concentrations of OM extract (25, 50, 100, 200 µg/ml) or RA (5, 10, 20 µg/ml) for 24 h prior to 100 µM H_2_O_2_ (IC_50_) stimulation were protected against H_2_O_2_-induced cytotoxicity (Fig. 2) (*p* < 0.001).

### Effects of OM extract on cognition

**Fig. 3:**
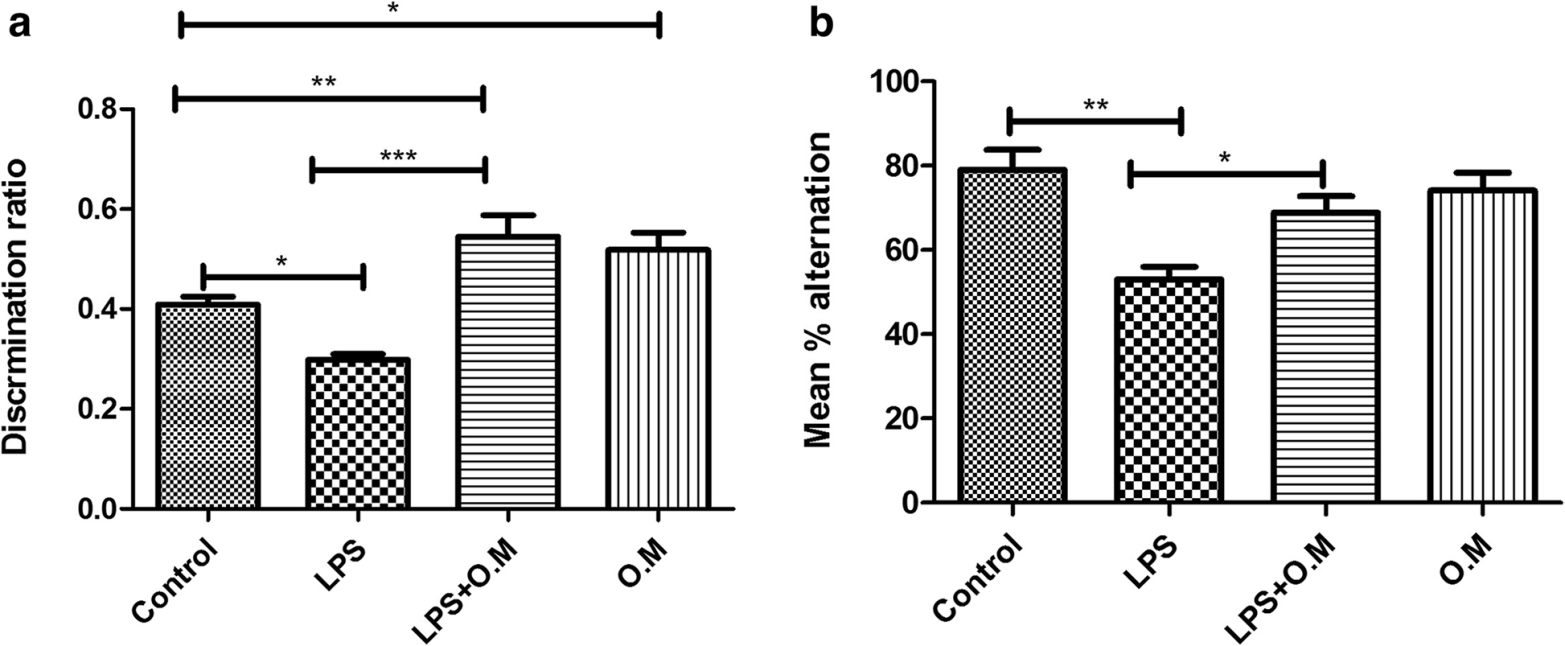
Effect of *Origanum majorana* on cognitive functions. LPS administration affected mean discrimination ratio (**a**) and alternation percentage (**b**). LPS + OM showed a significant increase in the mean discrimination ratio and alternation percentage compared to the LPS group. Mean discrimination ratio of LPS + OM and OM groups was higher than the control group. Data are expressed as mean ± SEM (*n* = 8), *P ≤ 0.05, **P ≤ 0.01, ***P ≤ 0.001

A significant deterioration of cognitive functions was found in mice injected with LPS where both the mean discrimination ratio (0.298 ± 0.011) (*p* < 0.05) and mean alternation percentage (53.03 ± 3.032) (*p* < 0.01) were significantly decreased compared to the controls (0.408 ± 0.016) and (79.00 ± 4.771), respectively. OM pre-treatment prevented the LPS-induced cognitive decline, as evaluated by the object recognition test (*p* < 0.001) and the Y-maze test (*p* < 0.05.), when compared to LPS group (0.545 ± 0.042) and (68.87 ± 3.901), respectively. OM Pre-treatment increased the discrimination ratio to higher than the control group (*p* < 0.01). The group receiving only OM extract also showed a higher discrimination ratio (0.518 ± 0.034) than the control group (*P* < 0.05) (Fig. 3).

### Impact of OM extract on LPS-induced neurodegeneration

**Fig. 4:**
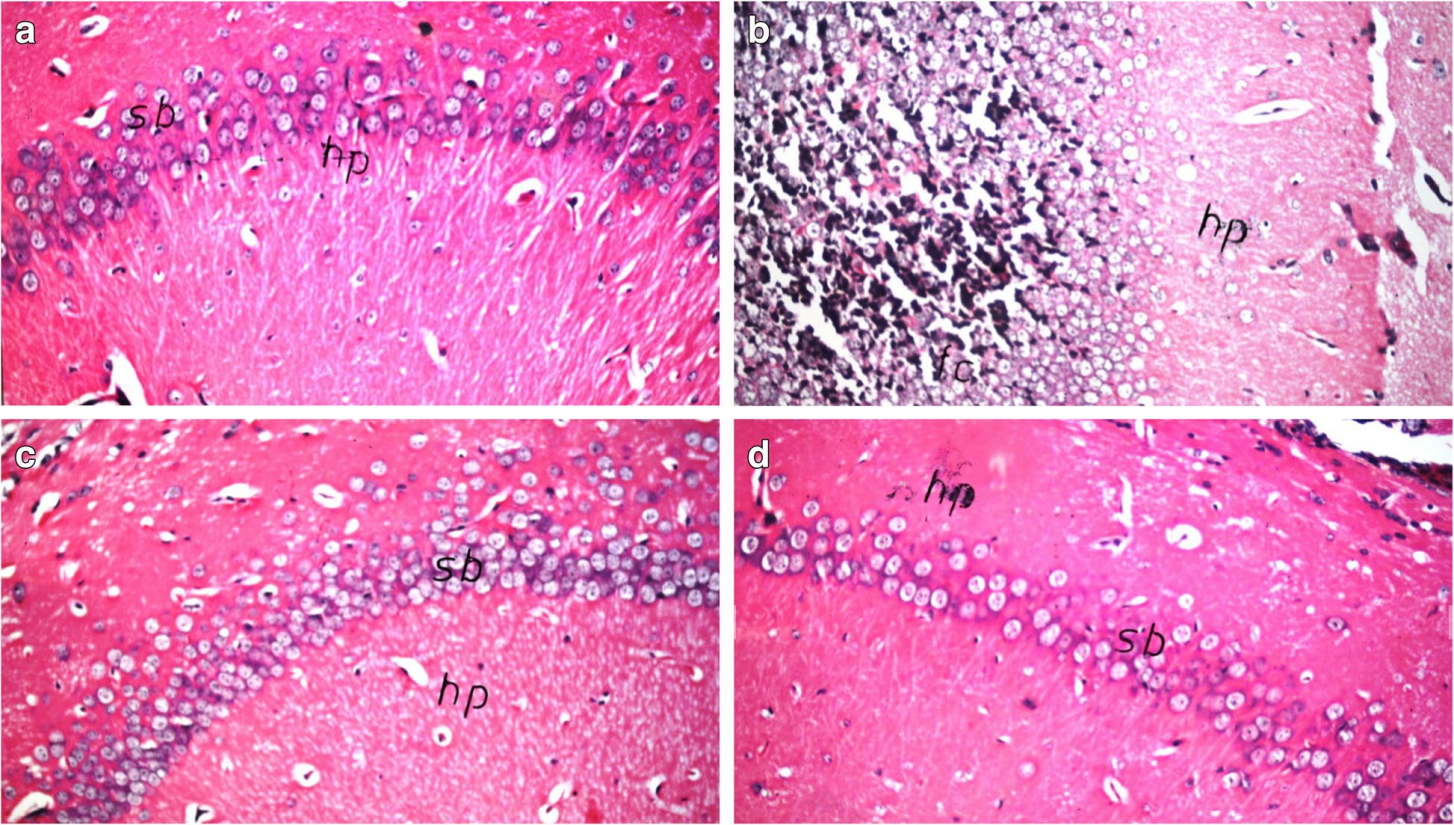
Photomicrograph of the hippocampus stained with H&E (× 40). LPS group (**b**) shows nuclear pyknosis and degeneration of the neurons compared to the control group (**a**). Pre-treatment with OM (**c**) shows comparable histology to the control group. OM (**d**) group presents normal histology (*n* = 3)

Hippocampal histopathological examination of LPS group reveals LPS-induced neuronal degeneration. Pre-treatment with OM in LPS + OM group showed comparable histology to the normal control group. No histological changes were observed in mice receiving OM extract only (Fig. 4). Similarly, striatum and cerebral cortex presented comparable histological features (data not shown).

### Modulatory effect of OM extract on astrocyte activation

Immunohistochemistry was performed to evaluate the astrocyte activation in terms of GFAP expression. Groups’ comparison was done by the semi-quantitative IHC profiler score and the IHC optical density score calculation, respectively.

**Fig. 5:**
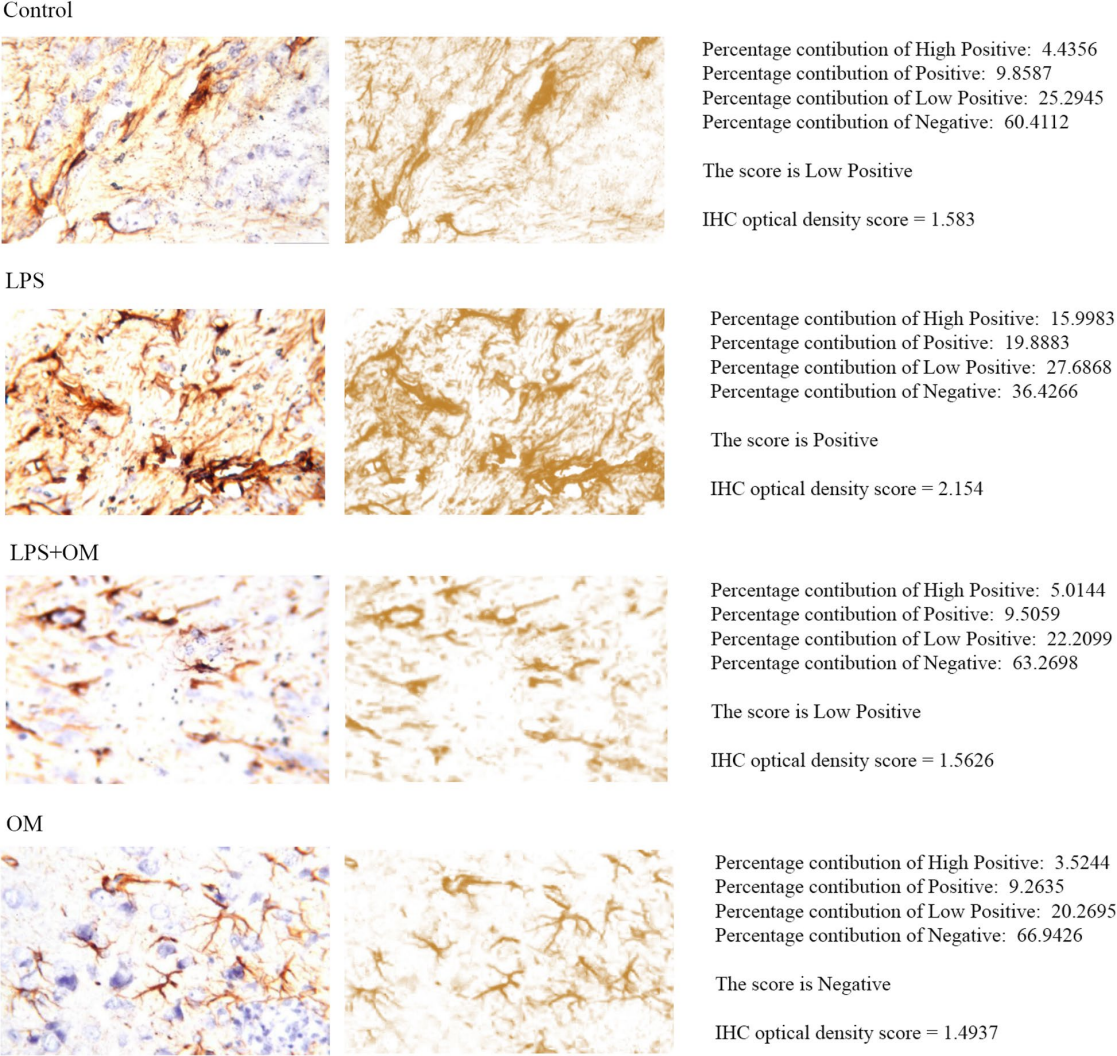
Immunohistochemical analysis of GFAP in the cerebral cortex. Deconvoluted images of DAB staining (second row) was produced by IHC profiler, and the percentage contributions was calculated relative to each image pixel intensity as high positive, positive, low positive, and negative pixels. Automated score of low positive is found for the control group and LPS + OM group, while LPS group presented as positive. The generated automated score for OM group is negative. For further quantitative analysis, calculation of IHC optical density score was done. LPS group scored 2.154 compared with 1.583 for control group, 1.5626 for LPS + OM group, and 1.4937 for OM group. Our results show that GFAP expression was enhanced in the LPS model and was attenuated under the effect of OM extract (COX-2 immunostaining, × 80) (*n* = 3)

LPS group exhibits higher GFAP immunoreactivity (Positive, 2.154) when compared to the control (Low Positive, 1.583). OM pre-treatment reduced GFAP expression (Low Positive, 1.5626) when compared to LPS group. OM presented a GFAP expression of 1.4937 (negative) (Fig. 5).

### Modulatory effect of OM extract on neuroinflammation

**Fig. 6:**
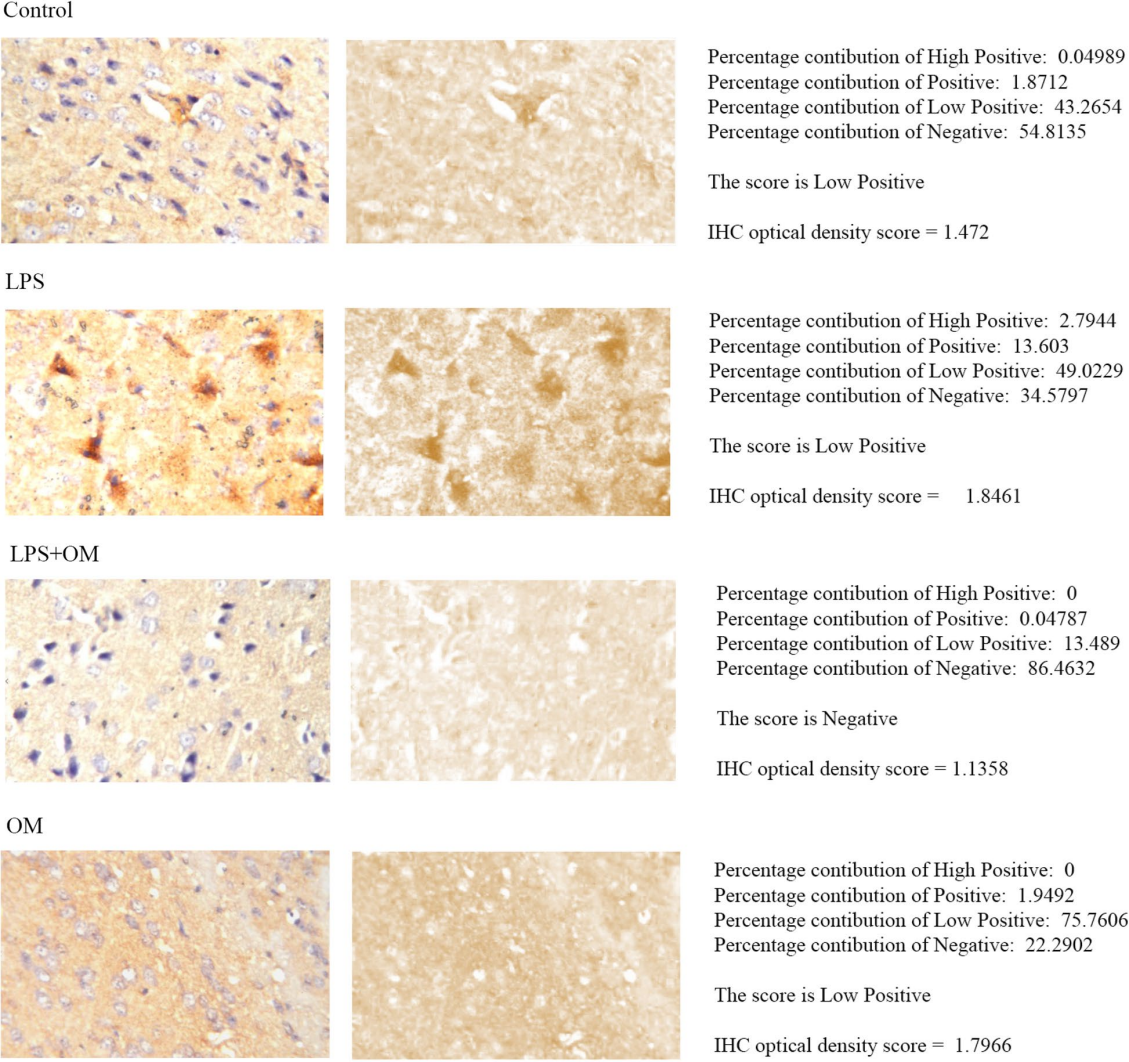
Immunohistochemical analysis of COX-2 in the cerebral cortex. Deconvoluted images of DAB staining (second row) was produced by IHC profiler, and the percentage contributions was calculated relative to each image pixel intensity as high positive, positive, low positive, and negative pixels. An automated score of low positive is found for the control group, LPS group, and OM group. However, the generated automated score for LPS + OM group is negative. For further quantitative analysis, calculation of IHC optical density score was done. LPS group scored 1.8461 compared with 1.472 for control group, 1.1358 for LPS + OM group, and 1.7966 for OM group. Our results show that COX-2 expression was attenuated under the effect of OM extract (COX-2 immunostaining, × 80) (*n* = 3)

COX-2 protein in the cerebral cortex was assessed using the same method adopted for GFAP analysis. LPS group presented a slight elevation in COX-2 expression (Low Positive, 1.846) when compared to control group (Low Positive, 1.472). OM pre-treatment, prior to LPS, decreased COX-2 expression (Negative, 1.135). The IHC profiler score of OM group was found in a range similar to the control group (Low Positive) (Fig. 6).

## Discussion

Strategies aiming to modulate the sustained oxidative stress- and inflammation-related insults on the CNS could mitigate the progression of neurodegenerative diseases. Natural dietary polyphenols, known to have antioxidants and anti-inflammatory properties, have gained increasing attention in recent decades for their beneficial effects on human health [53]. The edible plant *Origanum majorana* L. (OM) (also known as sweet marjoram) is native to the Mediterranean region and cultivated in Egypt, among other countries. Yet, it has not been sufficiently studied in the context of neurodegeneration and cognitive deterioration. A few studies showed that OM and *Origanum onites* L. (Turkish oregano) improved cognitive functions and memory in AD rat models with reported anti-acetylcholinesterase activities [54–56]. Moreover, hydroalcoholic extracts of OM have been shown to contain higher levels of phenolics and flavonoids than essential oils [57, 58], which encouraged us to assess the hydroalcoholic extract -rather than essential oils- of Egyptian OM in neuroinflammation-related neurodegeneration. Furthermore, a study conducted in Serbia found that among the ethanolic extracts of OM obtained from different countries, ethanolic extract of the Egyptian OM showed the highest phenolic and flavonoid contents with the highest level of rosmarinic acid [24]. In fact, the metabolic profiling of OM hydroalcohol extract, using HPLC/PDA/ESI/MS^n^, identified 17 compounds in our hands with a predominance of phenolics where rosmarinic acid (RA) and its derivatives represent most of the constituents with sagerinic acid and salvianolic being the major compounds. The antioxidant effect of RA has been further demonstrated in this study by protecting brain microglial cells against oxidative stress-mediated death. In the current study, a neuroinflammatory mouse model induced by LPS injected i.p. for seven consecutive days was used and characterized by a deterioration of the recognition memory associated with neurodegeneration and neuroinflammation as reflected by elevated COX-2 expression and astrocyte activation, reflected by an increased GFAP expression. The same model was previously characterized also by the buildup of Aβ protein 1–42 in the hippocampus and the cerebral cortex and an increased expression of inflammatory proteins such as inducible nitric oxide synthase (iNOS) and COX-2 in mice brains [17, 39], thus mimicking several pathophysiological aspects of AD with a prominent contribution of oxidative stress and inflammation. The treatment plan adopted in our study aimed at assessing the protective effect of OM hydroalcoholic extract by the daily administration of the extract prior to LPS and its continuation throughout the week of LPS injections. This approach goes along with strategies aiming to delay the onset of neurodegenerative diseases or slow their progression by mainly protecting the CNS against oxidative stress and inflammation. OM pre-treatment significantly protected against LPS-induced memory and cognitive impairment, as shown by the behavioral tests, which is most probably related to the attenuating effect of OM on the LPS-induced neurodegeneration that was observed upon histopathological examination of the brain tissue. The decreased COX-2 and GFAP expression in brain tissue of mice pre-treated with OM indicate the beneficial effect of this extract in attenuating the activation of pro-inflammatory pathways and the reactive astrogliosis, thus mitigating the inflammation-related neuronal death that is ultimately reflected in the protection against LPS-induced cognitive deterioration. To the best of our knowledge, the effect of OM hydroalcoholic extract on brain COX-2 and GFAP expression has not been previously evaluated in vivo. Extracts administered orally are subjected to pre-systemic metabolism in the digestive tract, which could significantly alter the phytochemical composition and, consequently their physiological behavior. For instance, RA intestinal permeability is limited, and only a little unchanged RA appears in the systemic plasma after being orally administered [59]. Thus, the systemic administration of OM extract adopted in this study makes it more feasible to link the effects observed in our model to previously reported properties of at least the major compounds identified in this extract. RA was reported to attenuate the TLR4/NF-κB (nuclear factor-kappaB) [60] and the GFAP immunoreactivity, protecting against memory deficits [61]. Salvianolic acid derivatives improved cognitive function and reduced inflammation by preventing cerebral NF-κB p65 activation in vascular dementia and cerebral ischemia reperfusion injury [62, 63]. Quercetin decreased the activation of glial cells in the hippocampus upon LPS injection in AD models, with a decrease in Aβ deposition [64]. Additionally, a previous study using the same LPS model found that quercetin could improve the memory, halt the LPS-induced activation of TLR4/NF-κB pathway and attenuate COX-2 expression [65]. Although these studies investigated isolated compounds rather than the whole extract, they offer a possible explanation of the protective effects of OM extract observed in the current study. Yet, the bioavailability of OM major active compounds in the brain tissue depends on their possible in vivo biotransformation and capability to pass the blood–brain barrier (BBB). For instance, while quercetin was shown to cross the BBB [66], the passage of RA to the brain was restricted by the BBB [59] in normal mice, which needs to be also verified in inflammatory models since peripheral cytokines might contribute to triggering BBB disruption and cognitive impairment [67].

## Conclusions

In conclusion, the outcomes of this study demonstrate that OM phenolic extract has the potential to protect against neurodegenerative diseases such as Alzheimer’s disease through targeting neuroinflammation, neurodegeneration, cognitive impairment, glial cell activation, and oxidative stress. Future studies still need to identify the major components responsible for these protective effects and decipher their mechanisms on the molecular level.

## Data Availability

The datasets used and/or analyzed during the current study are available from the corresponding author upon reasonable request.

## Abbreviations

AD: Alzheimer’s disease
Aβ: Amyloid beta
BBB: Blood–brain Barrier
COX: Cyclooxygenase
DAB: 3,3'-Diaminobenzidine
DAMP: Danger-associated molecular patterns
ESI: Electron Spray Ionization
FT-ICR: Fourier Transform Ion Cyclotron Resonance
GFAP: Glial fibrillary acidic protein
H_2_O_2_: Hydrogen peroxide
HPLC: High Pressure Liquid Chromatography
HPLC/PDA/ESI/MS^n^: High Performance Liquid Chromatography/ Photodiode Array Electrospray Ionization- Mass Spectrometry
H&E: Hematoxylin and Eosin
IHC: Immunohistochemistry
iNOS: Inducible nitric oxide synthase
i.p.: Intraperitoneal injection
IL: Interleukin
IL-6: Interleukin-60
LPS: Lipopolysaccharide
MS: Mass Spectrometry
NF-Κb: Nuclear factor-kappaB
OM: *Origanum majorana*
PDA: Photodiode array
RPMI: Roswell Park Memorial Institute medium
TLR: Toll-like receptor
TNF-α: Tumor necrosis factor alpha
WST-1: Water-soluble tetrazolium salt

## References

[1] Zhang B, Gaiteri C, Bodea L-G (2013). Integrated Systems Approach Identifies Genetic Nodes and Networks in Late-Onset Alzheimer’s Disease. Cell.

[2] Griffin WS, Stanley LC, Ling C (1989). Brain interleukin 1 and S-100 immunoreactivity are elevated in Down syndrome and Alzheimer disease. Proc Natl Acad Sci.

[3] Sheng JG, Mrak RE, Griffin WS (1997). Glial-neuronal interactions in Alzheimer disease: progressive association of IL-1alpha+ microglia and S100beta+ astrocytes with neurofibrillary tangle stages. J Neuropathol Exp Neurol.

[4] Lyra e Silva NM, Gonçalves RA, Pascoal TA, et al. Pro-inflammatory interleukin-6 signaling links cognitive impairments and peripheral metabolic alterations in Alzheimer’s disease. Transl Psychiatry. 2021;11:251. 10.1038/s41398-021-01349-z. 10.1038/s41398-021-01349-z PMC8080782 33911072

[5] Álvarez A, Cacabelos R, Sanpedro C (2007). Serum TNF-alpha levels are increased and correlate negatively with free IGF-I in Alzheimer disease. Neurobiol Aging.

[6] Motta M, Imbesi R, Di Rosa M (2007). Altered plasma cytokine levels in Alzheimer’s disease: Correlation with the disease progression. Immunol Lett.

[7] Millington C, Sonego S, Karunaweera N (2014). Chronic Neuroinflammation in Alzheimer’s Disease: New Perspectives on Animal Models and Promising Candidate Drugs. Biomed Res Int.

[8] Sheng WS, Hu S, Feng A, Rock RB (2013). Reactive Oxygen Species from Human Astrocytes Induced Functional Impairment and Oxidative Damage. Neurochem Res.

[9] Carter SF, Schöll M, Almkvist O (2012). Evidence for Astrocytosis in Prodromal Alzheimer Disease Provided by 11 C-Deuterium-L-Deprenyl: A Multitracer PET Paradigm Combining 11 C-Pittsburgh Compound B and 18 F-FDG. J Nucl Med.

[10] Muramori F, Kobayashi K, Nakamura I. A quantitative study of neurofibrillary tangles, senile plaques and astrocytes in the hippocampal subdivisions and entorhinal cortex in Alzheimer’s disease, normal controls and non-Alzheimer neuropsychiatric diseases. Psychiatry Clin Neurosci. 1998;52:593–9. 10.1111/j.1440-1819.1998.tb02706.x. 10.1111/j.1440-1819.1998.tb02706.x 9895207

[11] Miron J, Picard C, Frappier J (2018). TLR4 Gene Expression and Pro-Inflammatory Cytokines in Alzheimer’s Disease and in Response to Hippocampal Deafferentation in Rodents. J Alzheimer’s Dis.

[12] Balducci C, Frasca A, Zotti M (2017). Toll-like receptor 4-dependent glial cell activation mediates the impairment in memory establishment induced by β-amyloid oligomers in an acute mouse model of Alzheimer’s disease. Brain Behav Immun.

[13] Calvo-Rodriguez M, García-Rodríguez C, Villalobos C, Núñez L. Role of Toll Like Receptor 4 in Alzheimer’s Disease. Front Immunol. 2020;11. 10.3389/fimmu.2020.01588. 10.3389/fimmu.2020.01588 PMC7479089 32983082

[14] Zhao Y, Jaber V, Lukiw WJ. Secretory Products of the Human GI Tract Microbiome and Their Potential Impact on Alzheimer’s Disease (AD): Detection of Lipopolysaccharide (LPS) in AD Hippocampus. Front Cell Infect Microbiol. 2017;7. 10.3389/fcimb.2017.00318. 10.3389/fcimb.2017.00318 PMC5504724 28744452

[15] Miyake K (2007). Innate immune sensing of pathogens and danger signals by cell surface Toll-like receptors. Semin Immunol.

[16] Batista CRA, Gomes GF, Candelario-Jalil E (2019). Lipopolysaccharide-Induced Neuroinflammation as a Bridge to Understand Neurodegeneration. Int J Mol Sci.

[17] Lee J, Lee Y, Yuk D (2008). Neuro-inflammation induced by lipopolysaccharide causes cognitive impairment through enhancement of beta-amyloid generation. J Neuroinflammation.

[18] Zakaria A, Hamdi N, Abdel-Kader RM (2016). Methylene Blue Improves Brain Mitochondrial ABAD Functions and Decreases Aβ in a Neuroinflammatory Alzheimer’s Disease Mouse Model. Mol Neurobiol.

[19] Mostafa AO, Abdel-Kader RM, Heikal OA (2018). Enhancement of cognitive functions by rice bran extract in a neuroinflammatory mouse model via regulation of PPARγ. J Funct Foods.

[20] Wagdy R, Abdelkader RM, El-Khatib AH (2019). Neuromodulatory Activity of Dietary Phenolics Derived from Corchorus olitorius L. J Food Sci.

[21] Silva RFM, Pogačnik L (2020). Polyphenols from Food and Natural Products: Neuroprotection and Safety. Antioxidants.

[22] Dhull SB, Kaur P, Purewal SS (2016). Phytochemical analysis, phenolic compounds, condensed tannin content and antioxidant potential in Marwa (Origanum majorana) seed extracts. Resour Technol.

[23] Guerra-Boone L, Alvarez-Román R, Alvarez-Román R (2015). Antimicrobial and antioxidant activities and chemical characterization of essential oils of Thymusvulgaris, Rosmarinus officinalis, and Origanum majorana from northeastern México. Pak J Pharm Sci.

[24] Duletić-Laušević S, Aradski AA, Kolarević S, et al. Antineurodegenerative, antioxidant and antibacterial activities and phenolic components of Origanum majorana L. (Lamiaceae) extracts. J Appl Bot Food Qual. 2018;126–34. 10.5073/JABFQ.2018.091.018.

[25] Mir RH, Sawhney G, Verma R (2021). Origanum vulgare L.: In vitro Assessment of Cytotoxicity, Molecular Docking Studies, Antioxidant and Anti-inflammatory Activity in LPS Stimulated RAW 264.7 Cells. Med Chem (Los Angeles).

[26] Avola R, Granata G, Geraci C, et al. Oregano (Origanum vulgare L.) essential oil provides anti-inflammatory activity and facilitates wound healing in a human keratinocytes cell model. Food Chem Toxicol. 2020;144:111586. 10.1016/j.fct.2020.111586. 10.1016/j.fct.2020.111586 32679285

[27] Yoshino K, Higashi N, Koga K (2006). Antioxidant and Antiinflammatory Activities of Oregano Extract. J Heal Sci.

[28] Rababa’h AM, Alzoubi MA. Origanum majorana L. Extract Protects Against Isoproterenol-Induced Cardiotoxicity in Rats. Cardiovasc Toxicol. 2021;21:543–52. 10.1007/s12012-021-09645-2. 10.1007/s12012-021-09645-2 33786740

[29] Wahby MM, Yacout G, Kandeel K, Awad D (2015). LPS-induced oxidative inflammation and hyperlipidemia in male rats: The protective role of Origanum majorana extract. Beni-Suef Univ J Basic Appl Sci.

[30] Baby Sitty M, Chadalavada Vi. Anti-Parkinson’s activity and in vitro antioxidant activity of Origanum majorana plant extract. J Res Pharm. 2022;26(6):1814–24. 10.29228/jrp.272.

[31] Hassanzadeh-Taheri M, Ahmadi-Zohan A, Mohammadifard M, Hosseini M. Rosmarinic acid attenuates lipopolysaccharide-induced neuroinflammation and cognitive impairment in rats. J Chem Neuroanat. 2021;117:102008. 10.1016/j.jchemneu.2021.102008. 10.1016/j.jchemneu.2021.102008 34314849

[32] Handoussa H, Hanafi R, Eddiasty I (2013). Anti-inflammatory and cytotoxic activities of dietary phenolics isolated from Corchorus olitorius and Vitis vinifera. J Funct Foods.

[33] Elshamy S, Abdel Motaal A, Abdel-Halim M, et al. Potential neuroprotective activity of Mentha longifolia L. in aluminum chloride‐induced rat model of Alzheimer’s disease. J Food Biochem. 2021;45:1770. 10.1111/jfbc.13644. 10.1111/jfbc.13644 33587299

[34] Blainski A, Lopes G, de Mello J (2013). Application and Analysis of the Folin Ciocalteu Method for the Determination of the Total Phenolic Content from Limonium Brasiliense L. Molecules.

[35] Schofield P, Mbugua D, Pell A (2001). Analysis of condensed tannins: a review. Anim Feed Sci Technol.

[36] Zuo C-L, Wang C-M, Liu J (2018). Isoflurane anesthesia in aged mice and effects of A1 adenosine receptors on cognitive impairment. CNS Neurosci Ther.

[37] Cartner SC, Barlow SC, Ness TJ (2007). Loss of cortical function in mice after decapitation, cervical dislocation, potassium chloride injection, and CO2 inhalation. Comp Med.

[38] Aguwa US, Eze CE, Obinwa BN, et al. Comparing the Effect of Methods of Rat Euthanasia on the Brain of Wistar Rats: Cervical Dislocation, Chloroform Inhalation, Diethyl Ether Inhalation and Formalin Inhalation. J Adv Med Med Res. 2020;8–16. 10.9734/jammr/2020/v32i1730636.

[39] Khan MS, Ali T, Kim MW (2016). Anthocyanins protect against LPS-induced oxidative stress-mediated neuroinflammation and neurodegeneration in the adult mouse cortex. Neurochem Int.

[40] Hassaan Y, Handoussa H, El-Khatib AH (2014). Evaluation of Plant Phenolic Metabolites as a Source of Alzheimer’s Drug Leads. Biomed Res Int.

[41] Lee B, Shim I, Lee H (2018). Gypenosides Attenuate Lipopolysaccharide-Induced Neuroinflammation and Memory Impairment in Rats. Evidence-Based Complement Altern Med.

[42] Bevins RA, Besheer J (2006). Object recognition in rats and mice: a one-trial non-matching-to-sample learning task to study “recognition memory”. Nat Protoc.

[43] Arai K, Matsuki N, Ikegaya Y, Nishiyama N (2001). Deterioration of Spatial Learning Performances in Lipopolysaccharide-Treated Mice. Jpn J Pharmacol.

[44] Varghese F, Bukhari AB, Malhotra R, De A. IHC Profiler: An Open Source Plugin for the Quantitative Evaluation and Automated Scoring of Immunohistochemistry Images of Human Tissue Samples. PLoS One. 2014;9:e96801. 10.1371/journal.pone.0096801. 10.1371/journal.pone.0096801 PMC4011881 24802416

[45] Ye M, Yang W-Z, Liu K-D (2012). Characterization of flavonoids in Millettia nitida var. hirsutissima by HPLC/DAD/ESI-MSn. J Pharm Anal.

[46] El Sayed AM, Ezzat SM, El Naggar MM, El Hawary SS (2016). In vivo diabetic wound healing effect and HPLC–DAD–ESI–MS/MS profiling of the methanol extracts of eight Aloe species. Rev Bras Farmacogn.

[47] Barros L, Dueñas M, Dias MI (2013). Phenolic profiles of cultivated, in vitro cultured and commercial samples of Melissa officinalis L. infusions. Food Chem.

[48] Chen H-J, Inbaraj BS, Chen B-H (2011). Determination of Phenolic Acids and Flavonoids in Taraxacum formosanum Kitam by Liquid Chromatography-Tandem Mass Spectrometry Coupled with a Post-Column Derivatization Technique. Int J Mol Sci.

[49] Nyau V, Prakash S, Rodrigues J, Farrant J (2015). Idenfication of Nutraceutical Phenolic Compounds in Bambara Groundnuts (Vigna subterranea L. Verdc) by HPLC-PDA-ESI-MS. Br J Appl Sci Technol.

[50] Simirgiotis M, Benites J, Areche C, Sepúlveda B (2015). Antioxidant Capacities and Analysis of Phenolic Compounds in Three Endemic Nolana Species by HPLC-PDA-ESI-MS. Molecules.

[51] Sakalem ME, Negri G, Tabach R (2012). Chemical composition of hydroethanolic extracts from five species of the Passiflora genus. Rev Bras Farmacogn.

[52] Simirgiotis M (2013). Antioxidant Capacity and HPLC-DAD-MS Profiling of Chilean Peumo (Cryptocarya alba) Fruits and Comparison with German Peumo (Crataegus monogyna) from Southern Chile. Molecules.

[53] Cory H, Passarelli S, Szeto J, et al. The Role of Polyphenols in Human Health and Food Systems: A Mini-Review. Front Nutr. 2018;5. 10.3389/fnut.2018.00087. 10.3389/fnut.2018.00087 PMC6160559 30298133

[54] Mossa ATH, Nawwar G (2011). Free radical scavenging and antiacetylcholinesterase activities of Origanum majorana L. essential oil. Hum Exp Toxicol.

[55] Postu PA, Gorgan DL, Cioanca O (2020). Memory-Enhancing Effects of Origanum majorana Essential Oil in an Alzheimer’s Amyloid beta1-42 Rat Model: A Molecular and Behavioral Study. Antioxidants.

[56] Aykac A, Teralı K, Özbeyli D (2022). A multi-parameter evaluation of the neuroprotective and cognitive-enhancing effects of Origanum onites L. (Turkish Oregano) essential oil on scopolamine-induced amnestic rats. Metab Brain Dis.

[57] Bina F, Rahimi R (2017). Sweet Marjoram. J Evid Based Complementary Altern Med.

[58] Semiz G, Semiz A, Mercan-Doğan N (2018). Essential oil composition, total phenolic content, antioxidant and antibiofilm activities of four Origanum species from southeastern Turkey. Int J Food Prop.

[59] Hase T, Shishido S, Yamamoto S (2019). Rosmarinic acid suppresses Alzheimer’s disease development by reducing amyloid β aggregation by increasing monoamine secretion. Sci Rep.

[60] Wei Y, Chen J, Hu Y (2018). Rosmarinic Acid Mitigates Lipopolysaccharide-Induced Neuroinflammatory Responses through the Inhibition of TLR4 and CD14 Expression and NF-κB and NLRP3 Inflammasome Activation. Inflammation.

[61] Fonteles AA, de Souza CM, de Sousa Neves JC (2016). Rosmarinic acid prevents against memory deficits in ischemic mice. Behav Brain Res.

[62] Zhang W, Song J-K, Zhang X, et al. Salvianolic acid A attenuates ischemia reperfusion induced rat brain damage by protecting the blood brain barrier through MMP-9 inhibition and anti-inflammation. Chin J Nat Med. 2018;16:184–93. 10.1016/S1875-5364(18)30046-3. 10.1016/S1875-5364(18)30046-3 29576054

[63] Ma X, Xu W, Zhang Z (2017). Salvianolic Acid B Ameliorates Cognitive Deficits Through IGF-1/Akt Pathway in Rats with Vascular Dementia. Cell Physiol Biochem.

[64] Sabogal-Guáqueta AM, Muñoz-Manco JI, Ramírez-Pineda JR (2015). The flavonoid quercetin ameliorates Alzheimer’s disease pathology and protects cognitive and emotional function in aged triple transgenic Alzheimer’s disease model mice. Neuropharmacology.

[65] Khan A, Ali T, Rehman SU, et al. Neuroprotective Effect of Quercetin Against the Detrimental Effects of LPS in the Adult Mouse Brain. Front Pharmacol. 2018;9. 10.3389/fphar.2018.01383. 10.3389/fphar.2018.01383 PMC6297180 30618732

[66] Li Y, Zhou S, Li J (2015). Quercetin protects human brain microvascular endothelial cells from fibrillar β-amyloid1–40-induced toxicity. Acta Pharm Sin B.

[67] Geng J, Wang L, Zhang L, et al. Blood-Brain Barrier Disruption Induced Cognitive Impairment Is Associated With Increase of Inflammatory Cytokine. Front Aging Neurosci. 2018;10. 10.3389/fnagi.2018.00129. 10.3389/fnagi.2018.00129 PMC5949351 29867440

